# The Effect of the Photoperiod on the Fatty Acid Profile and Weight in Hatchery-Reared Underyearlings and Yearlings of Atlantic Salmon *Salmo salar* L.

**DOI:** 10.3390/biom10060845

**Published:** 2020-06-02

**Authors:** Nina N. Nemova, Zinaida A. Nefedova, Svetlana N. Pekkoeva, Viktor P. Voronin, Natalia S. Shulgina, Maria V. Churova, Svetlana A. Murzina

**Affiliations:** Environmental Biochemistry Lab, Institute of Biology of the Karelian Research Centre of the Russian Academy of Sciences, Pushkinskaya st., 11, 185910 Petrozavodsk, Russia; znefed@krc.karelia.ru (Z.A.N.); pek-svetlana@mail.ru (S.N.P.); voronen-viktor@mail.ru (V.P.V.); shulgina28@yandex.ru (N.S.S.); mchurova@yandex.ru (M.V.C.)

**Keywords:** fatty acids, total lipids, photoperiod, Atlantic salmon *Salmo salar* L., aquaculture

## Abstract

The influence of two light regimes, 16:8 h light/dark (LD 16:8) and 24:0 h light/dark (LD 24:0), in comparison to a usual hatchery light regime (HL), on the fatty acids content and weight gain in hatchery-reared underyearlings (at 0+ age) and yearlings (at 1+ age) of Atlantic salmon in the summer–autumn period was studied. The total lipids were analyzed by Folch method, the lipid classes using HPTLC, and the fatty acids of total lipids using GC. The increase in EPA and DHA observed in October in underyearlings and yearlings salmon (especially under LD 24:0) suggests they were physiologically preparing for overwintering. The changes in fatty acids and their ratios in juvenile Atlantic salmon can be used as biochemical indicators of the degree to which hatchery-reared fish are ready to smoltify. These associated with an increase in marine-type specific DHA and EPA, an increase in the 16:0/18:1(n-9) ratio, in correlation with a reduction in MUFAs (mainly 18:1(n-9)). These biochemical modifications, accompanied by fish weight gain, were more pronounced in October in yearlings exposed to continuous light (LD 24:0). The mortality rate was lower in experimental groups of underyearliings with additional lighting. Exposure to prolonged and continuous light did not affect yearlings mortality rate.

## 1. Introduction

Many physiological and biochemical processes in fish undergo daily fluctuations (circadian rhythms), and their activity is governed by the endocrine system [1]. For instance, the growth hormone content in Atlantic salmon (*Salmo salar* L.) smolts increases as light duration is prolonged [2]. Various photoperiod modes trigger the parr–smolt transformation and smoltification, which is associated with elevated endocrine and Na^+^,K^+^-ATPase activity [3]; increase in lipid and nitrogen metabolism [4]; changes in the bioconversion of essential fatty acids (FAs) α-linoleic acid (18:3(n-3)) and linoleic acid (18:2(n-6)) into physiologically valuable polyunsaturated FAs (PUFAs) eicosapentaenoic acid (EPA, 20:5(n-3)), docosahexaenoic acid (DHA, 22:6(n-3)), and arachidonic acid (ARA, 20:4(n-6)) [5,6,7,8]; and the growth of young salmon [9,10,11]. The fish response to light modes depends on the physiological condition of the organism: age, gut fullness, condition and fitness, reproductive status, and maturity, as well as water temperature [12]. The locomotor and foraging activity of salmon parr is constrained by light conditions (not less than 16,000 and not more than 80,500 lx) [13]. For wild salmon juveniles [8], lipid metabolism indices are quite sensitive to changes in the ambient environment. This involves modifications in structural lipids and their fatty acid components to ensure the optimal viscosity of the biomembrane lipid bilayer and, thus, normal functioning of membrane-bounded proteins and receptors [14]. Studying the lipid status in the summer–autumn period is important because this period is decisive for the physiological preparation of young salmonids for their first winter, and can influence the timing of smoltification and the life strategy [15]. 

Many physiological and biochemical processes in fish undergo daily fluctuations (circadian rhythms), and their activity is governed by the endocrine system [1]. For instance, the growth hormone content in Atlantic salmon (*Salmo salar* L.) smolts increases as light duration is prolonged [2]. Various photoperiod modes trigger the parr–smolt transformation and smoltification, which is associated with elevated endocrine and Na^+^,K^+^-ATPase activity [3]; increase in lipid and nitrogen metabolism [4]; changes in the bioconversion of essential fatty acids (FAs) α-linoleic acid (18:3(n-3)) and linoleic acid (18:2(n-6)) into physiologically valuable polyunsaturated FAs (PUFAs) eicosapentaenoic acid (EPA, 20:5(n-3)), docosahexaenoic acid (DHA, 22:6(n-3)), and arachidonic acid (ARA, 20:4(n-6)) [5,6,7,8]; and the growth of young salmon [9,10,11]. The fish response to light modes depends on the physiological condition of the organism: age, gut fullness, condition and fitness, reproductive status, and maturity, as well as water temperature [12]. The locomotor and foraging activity of salmon parr is constrained by light conditions (not less than 16,000 and not more than 80,500 lx) [13]. For wild salmon juveniles [8], lipid metabolism indices are quite sensitive to changes in the ambient environment. This involves modifications in structural lipids and their fatty acid components to ensure the optimal viscosity of the biomembrane lipid bilayer and, thus, normal functioning of membrane-bounded proteins and receptors [14]. Studying the lipid status in the summer–autumn period is important because this period is decisive for the physiological preparation of young salmonids for their first winter, and can influence the timing of smoltification and the life strategy [15]. 

In Russia, the population pool of Atlantic salmon constitutes a substantial part of the species globally. Nowadays, harvestable salmonid stocks in the north-western region are severely depleted and are primarily maintained by artificial breeding. Hatchery-reared young fish, in conditions of mixed photoperiod in Northwest Russia (natural light periods in the summer known as “white nights” and hatchery light for the rest of the year), raised until the age of two years for salmon are released into natural freshwater from where fish migrate to the sea. A high rate of return of harvestable salmonids can only be attained if hatcheries release the young physiologically ready for the foraging migration into the sea [16,17,18]. Smoltification is an important part of salmonid ontogeny and it is accompanied by complex physiological and biochemical changes. Moreover, the onset of salmon’s parr–smolt transformation depends on the size attained by the fish in the autumn before the year of smoltification [19,20,21]. Among factors of the physiological preparedness of the young for habitat change, size of youngs is a key one, since its increase means the approach of smoltification, and hence raises the adaptive potential and survival capacity of individuals. 

We studied the effect of different light regimes on growth and the content of fatty acids and it is ratios in hatchery-reared underyearlings (at 0+ age) and yearlings (at 1+ age) of Atlantic salmon in the summer–autumn period. The hypothesis is that the experimental prolonged and constant light has “stimulus” effect on the weight and lipid metabolism evaluated by the analysis of certain fatty acids including physiologically valuable EPA and DHA. 

## 2. Materials and Methods

### 2.1. Sample Collection and Description

The experiment was conducted at the Vygsky fish hatchery (Belomorskij region, Russia, 64°25′ N and 34°28′ E). The rearing tanks were located in the hatchery building with flow-through water supply. 

The duration of the experiment for underyearlings (0+) was 3 months, from August to October; as soon as fry reached a mass of 0.9 g, they were transferred from the incubation center (larval culture area) to the juvenile rearing tanks. After a week of adaptation to the new conditions, the experiment commenced (at the beginning of August). The effect of photoperiod on underyearlings was evaluated in September and in October (in August, the light regimes were installed and switched on). The average number of fingerlings were 7400 per tank. The average weight of the underyearlings in all groups at the beginning of the study was: 0.94 ± 0.03 g. The underyearlings were weighed several times every month. Data on fish weight were obtained from the results of the repeated weighing (five times) of 100 individuals together. These data were used in discussion of the effect of light regime on weight characteristics of underyearlings (0+). 

The duration of the experiment for yearlings (1+) was 4 months, from July to October. The effect of photoperiod on yearlings was evaluated in August, September and October. The fish exposed different light regimes from July; thus, the effect of photoperiod for yealings was discussed from August. The average number of fish was 1245 per tank. At the beginning of the study, the fish in each tank (160 ind./per pool) were tagged using PIT-tags (Felixcan SL; Felixcan, Spain). The PIT-tag injection was as follows: the fish were anesthetized with clove oil then medium-sized individuals within the weight range of 7–14 g were tagged. The average weight of the fish selected for observation in each group was the same and was 10.20 ± 0.13 g (group 1, HL), 10.21 ± 0.04 g (group 2, LD 16:8), 10.16 ± 0.15 g (group 3, LD 24:0). Every month, weight and length measurements of 40 tagged fish per group were assessed. These data were used in a discussion of the effect of light on weight characteristics of yearlings (1+).

**Figure 1:**
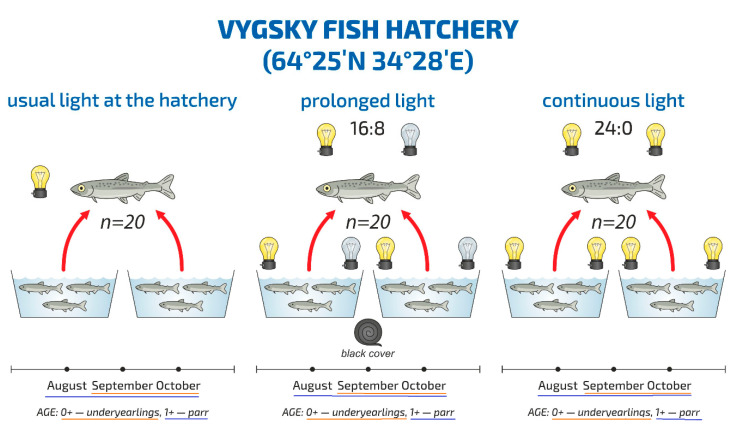
Scheme of the experiment at the Vygsky fish factory. Different photoperiod regimes: usual light at the hatchery, HL (hatchery lighting), the experimental regimes—prolonged light, LD 16:8, continuous light, LD 24:0—are described in detail in the text.

The experimental groups of fish were subjected to the following light regimes: prolonged light, light/dark (LD) 16:8 h; continuous light (LD 24:0). For each regime, two parallel tanks were taken and the fry were inside (Figure 1). 

The experimental tanks were equipped with two light-emitting diodes (LED) lights (Aquael leddy smart LED sunny, 6 W, 6500 K; Aquael, Warsaw, Poland) and placed on tanks’ wells, which were diagonal to each other and also covered with black, light-tight film. The lights were controlled by automatic timers that were preset to turn the power on and off at 6:00 a.m. and 10:00 p.m., respectively (Feron TM-50; Feron, Moskow, Russia). The light intensity was 760 lx at the water’s surface under the LED lights and 400 lx around it, 45 lx on the opposite side, and 70 lx in the middle of the tank. 

The usual light regime of the hatchery was used for results comparison (HL). HL groups were subjected to natural light from outside (the light came from the wall-size window) during July and the first half of August, and the light intensity at the water’s surface was 12 lx during the day and 2 lx during the night. Then, the hatchery’s light was turned on from 5:00 p.m. until 8:00 a.m. for 1 month. The lamps were located on the building’s walls and were positioned sideways. In Table 1, the time period and the light regime used is performed. The light intensity at the water’s surface at the center of the tank was 10 lx in the day and 8 lx in the night. Then, the light was turned on all the time starting 10 September. The light intensity at the water’s surface was uneven, with 8 lx in the center.

All other rearing conditions were the same: fish-holding density, feed and feeding regime, preventive measures, and care of the pools. The fish were fed a commercial feed according to the fish hatchery’s recommendations, which depended on water temperature fluctuations. The commercial feed for underyearlings (0+) Biomar Inicio plus G (Biomar, Danmark) contained 57–58% crude protein, 14%–18% crude lipid, and 8–10% carbohydrates, depending on pellet size. Commercial feed for yearlings Biomar Inicio 917 (Biomar, Danmark) was used. It contained 47–50% crude protein, 16–23% crude lipid, and 15.4–20.2% carbohydrates, depending on pellet size. The day ratio for underyearlings (0+) was 2.9–3.1% in August, 2.5–1.3–1.1% in September (according to decades), and 1.1% in October. The day ratio for yearlings (1+) was 1.8–1.5% in July, 1.3–2.0% in August, 1.2–0.9% in September, and 0.5% in October. The feeding regime was organized according to the General Rules and Recommendations at the hatchery and based on the temperature of the water (the factor influences the period of active growth of fish) and sustainable feeding of the hatchery-reared fish. Automatic feeders used to feed the hatchery-reared underyearlings and yearlings. Next parameters were programmed and automated for underyearlings: the feeding time was from 6 a.m. to 11 p.m., the frequency was 20–30 s in August; the feeding time was from 9 a.m. to 6 p.m., the frequency was 20–35 s in September and October. For yearlings: the feeding time was from 6 a.m. to 10 p.m. in July and August, and the frequency was 20–30 s; and the feeding time was from 9 a.m. to 6 p.m., and the frequency was 40–45 s in September and October. 

Water flowed into the tanks from the Matkozhnenskoe reservoir (Nizhnij Vyg River). The flow velocity was 40 L min^−1^ and 60 L min^−1^ for fish at 0+ and 1+ age, respectively. The temperature on the sampling day was 15.2 °C on July 9, 16.6 °C on August 8, 13.3 °C on September 5, and 8.2 °C on October 5. Temperature fluctuations during the study period were 14.8–19.9 °C in July, 18.2–13.8 °C in August, 13.8–9.8 °C in September, and 9.8 °C–2.4 °C in October.

The collection procedure was the same for underyearlings and yearlings (parr). From each HL tank, 10 fry were collected for lipid analysis. The total number was 20 specimens in the HL group. We selected 10 fry from each experimental tank under LD 16:8 and from each experimental tank under LD 24:0; the total number of fishes per group was 20. The flesh (white) muscle samples for lipid and fatty acid analysis were obtained every month to evaluate the effect of photoperiod (from September to October for underyearlings; from August to October for yearlings). The length-weight characteristics of the fish taken for lipid analysis are presented in Table 2 and Table 3.

The condition factor was calculated as CF (%) = W × 100/L^3^, where W is the weight of a fish, g, and L is the length of a fish. The mortality rate was calculated as S (%) = (number of dead fish/total fish) × 100. 

### 2.2. Lipid Extraction and Lipid Classes Analysis

The flesh of the studied fishes (the entire volume of muscles of underyearlings and yearlings) was homogenized in glass vials in chloroform/methanol (2:1, *v*/*v*) solution (10 mL per 1–3 g wet weight). The total lipids (TLs) were extracted using the Folch method [22]. The homogenate was filtered, and the residue retained on the paper filter was rinsed with 30 mL of the extractive mixture. Then, the extract was mixed by adding chloroform and deionized water, which was left to settle in a separatory glass funnel until the complete separation of organic phases. Lipids remain in the lower chloroform layer, while non-lipid substances move to the upper aqueous methanol phase. Then, the chloroform layer was withdrawn for evaporated in a vacuum on a rotary evaporator (Hei-VAP Advantage HL/G3, Heidolph, Schwabach, Germany), and dried in a vacuum over phosphoric anhydride to constant weight. The total lipids were dissolved in chloroform/methanol and stored at −20 °C. 

Qualitative and quantitative determination of individual lipid classes as total phospholipids (PL), di-, triacylglucerols (DAG, TAG respectively), cholesterol (Chol), sterol esters (mainly cholesterol esters) and free fatty acids (FFA) was carried out using the high-performance thin-layer chromatography (HPTLC). Fractionation of total lipids was performed on ultrapure glass HPTLC Silica gel 60 F_254_ Premium Purity plates (Merck, Germany). Spot 2 μL of the sample was sprayed onto HPTLC plates with a 100 μL dosage syringe for Linomat (CAMAG, Switzerland) by semi-automatic applicator Linomat 5 controlled by visionCATs HPTLC software (CAMAG, Switzerland) in the form of a narrow band 6 mm in length, 8 mm from the bottom. The solvent system of hexane/diethyl ether/acetic acid (32:8:0.8, by volume) [23] was used both as an eluent and as a solution for chromatographic chamber ADC 2 (CAMAG, Switzerland) saturation. Supersaturated zinc nitrate (ZnNO_3_*6H_2_O) solution was used for maintaining humidity in the chromatographic chamber (47–49% humidity). Chromatographic chamber saturation (20 min) and conditioning (10 min) were performed simultaneously, and then the plate was saturated for 20 min. The height of the mobile phase front was 80 mm (Rf = 80 mm). The plate was dried for 5 min. Formation of visible lipid spots was carried out in a solution of copper sulfate (CuSO_4_) and phosphoric acid (H_3_PO_4_) by heating the plate to 160 °C for 15 min. The determination of lipid classes was carried out on a deuterium lamp (wavelength of 350 nm) of a TLC Scanner 4 densitometer chamber (CAMAG, Switzerland) [24]. The identification of lipid classes was carried out according to the standards of the respective studied components (Sigma-Aldrich, USA; Avanti Polar Lipids, Inc., USA) taking into account correspondence of the Rf values.

### 2.3. Fatty Acid Analysis

The fatty acid profile of the total lipid extracts was analyzed by gas chromatography (GC). The methylation of fatty acids from the lipid extracts was performed in a glass retort in which 0.1 mL of a solution containing 20 mg/10 mL (behenic FA, C22:0; Sigma Aldrich, St. Louis, MO, USA) in methanol was added as the internal standard and the transesterification was performed in methanol (2 mL) containing chlorate acetyl (0.2 mL) at 70 °C for 90 min (using a Schott Duran glass serpentine condenser). After extraction, cooling with hexane was carried out in glass serpentine condensers rinsed with 5 mL of hexane for each sample. We added 2 mL of deionized water to each glass retort for phase separation in the separatory glass funnels for 15 min. Fatty acids methyl esters (FAME) remain in the upper hexane layer, whereas other substances move to the lower aqueous phase. The hexane layer was then withdrawn to be evaporated under a vacuum on a rotary evaporator Hei-VAP Advantage HL/G3 (Heidolph, Schwabach, Germany). Then, 0.9 mL hexane for GC (Sigma Aldrich, St. Louis, MO, USA) was added to each glass retort and the contents moved to glass GC vials for following GC analysis.

FAME were identified using a Chromatek-Crystall-5000.2 (Chromatek, city, Russia) gas chromatograph with a flame-ionization detector (FID) and a Zebron ZB-FFAP capillary gas chromatographic column (Phenomenex, Torrance, CA, USA). An isothermal column configuration was used (200 °C); the temperatures of the detector and evaporator were 250 and 240 °C, respectively. Chromatek-Analytik-5000.2 software (Chromatek, Yoshkar-Ola, Russia) was used for recording and integrating the data. FAMEs were identified with standard mixtures of Supelco 37 Component FAME mix, bacterial acid methyl ester (BAME), and PUFA No. 1 (Sigma Aldrich, St. Louis, MO, USA), and by comparing the equivalent lengths of carbon chains and table constants according to Jamieson [25].

The biochemical analysis was performed at the Scientific Center collective usage platform of the Karelian Research Centre of the Russian Academy of Sciences.

### 2.4. Statistical Analysis

To perform statistical analysis, the free R-programming language with basic packages and additional “tidyverse”, “psych”, “coin” packages was used. The data were analyzed to determine whether they exhibited a normal distribution. The significant differences in the means of the studied lipids and fatty acids between fish collected in different seasons and exposure to different photoperiod were tested by non-parametric test, the Wilcoxon-Mann-Whitney test from the “coin” package of R. Statistical significance was set at *p* ≤ 0.05.

## 3. Results

### 3.1. Weight Gain

**Figure 2:**
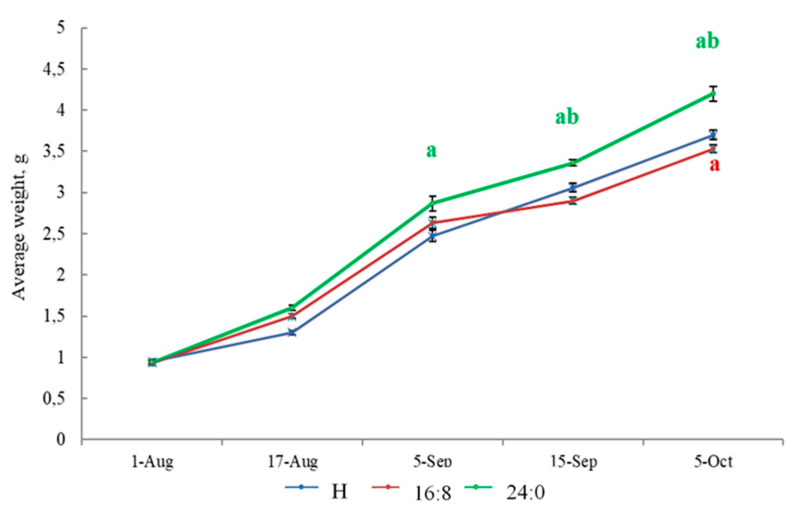
Average weight of underyearlings (at 0+ age) under different photoperiod regimes: HL (hatchery light regime), and the experimental regimes (LD 16:8, LD 24:0 from August 5 to October 5). The effect of photoperiod on underyearlings was evaluated in September and in October (in August, the light regimes were installed and switched on). Subscript letters mean: **a**—significant differences compared with the HL group at the day of assessment; **b**—significant differences compared with LD 16:8 regime at the day of assessment.

At the end of the experiment, the average mass of the underyearlings (0+) from group 3 (with constant light) significantly exceeded the mass of the fishes from groups 1 and 2 (*p* < 0.05) (Figure 2).

Differences in the mass of the fish from group 3 (LD 24:0) compared with group 1 (HL) appeared in the first month of the study and remained until the end of the study. The weight gain at the end of the experimental period was 2.75 g in group 1 (HL), 2.60 g in group 2 (LD 16:8) and 3.26 g in group 3 (LD 24:0) (*p* < 0.05). Noticeable, by the end of September, there was a decrease in the weight gain of the fish in the group 2 (LD 16:8), and in October, their mean mass was significantly lower than the fish in the HL group (Figure 2).

The mortality rate for underyerlings was 1.80% in group 1, 0.60% in group 2, and in group 3, 0.95%.

**Figure 3:**
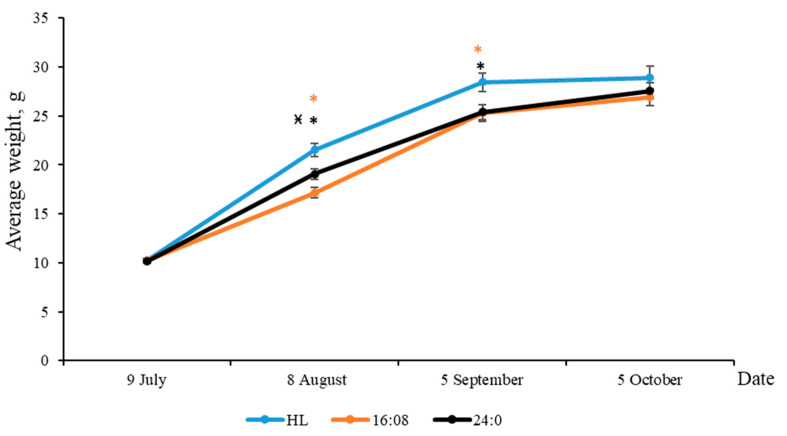
Average weight of yearlings (at 1+ age) under different photoperiod regimes: HL (hatchery light regime), and experimental regimes (LD 16:8, LD 24:0 from July 9 to October 5). The fish were exposed to different light regimes from July (fish were loaded into the tanks and the experiment was started); thus, the effect of photoperiod for yealings is discussed from August. Subscript signs: *—significant differences compared with the HL group at the day of assessment, *p* < 0.05; х—significant differences compared with LD 16:8 at the day of assessment, *p* < 0.05.

The yearlings’ (at 1+ age) weight was significantly lower in group 2 and group 3 in comparison to group 1 at the beginning of August and September (Figure 3).

In group 2, the weight of fish in August was lower in comparison to group 3 with constant light. It was observed that close to the end of the experiment, yearlings in groups 2 and 3 reached the weight of the fish in group 1. At the end of the experiment, the differences in the weight of individuals among groups were not established. The weight gains for the different groups from July to October were found as follows: group 1—18.70 g, group 2—16.66 g, group 3—17.40 g (Figure 3).

Exposure to prolonged and continuous light did not affect yearlings’ mortality rate: there were no dead yearlings during the study period.

### 3.2. Total Lipids and Lipid Classes

**Figure 4:**
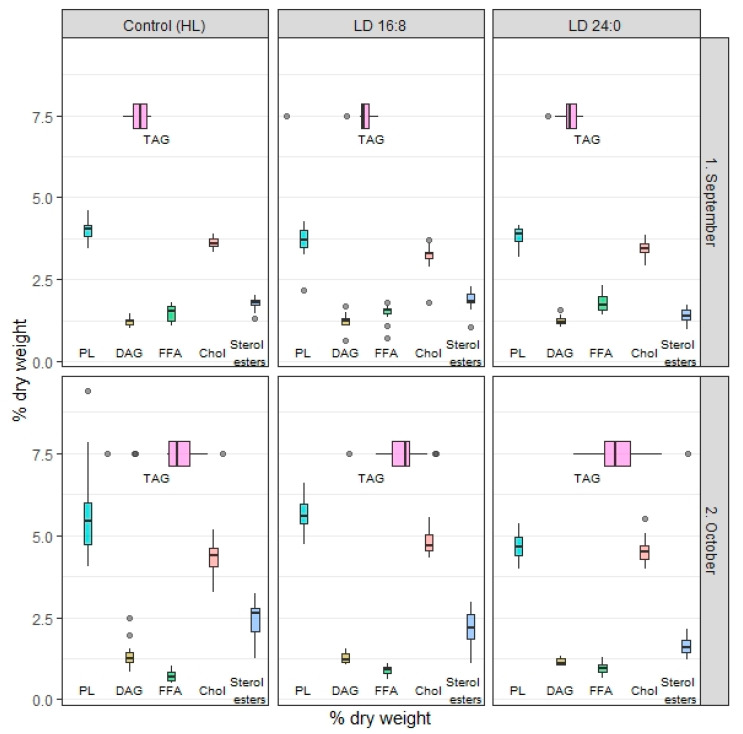
The changes in the lipid classes (% dry weight) in underyearlings (at 0+ age) of Atlantic salmon (*Salmo salar* L.) under different photoperiod regimes: HL (hatchery light), and the experimental regimes (LD 16:8, LD 24:0 from August 5 to October 5). The effect of photoperiod on underyearlings was evaluated in September and in October (in August, the light regimes were installed and switched on).

In September, TL content ranged from 26.6% to 27.1% dry weight and CF accounted for 1.1% in underyearlings exposed to different photoperiod regimes; no significant differences were found (Table 2). The contents of total PL, DAG, TAG, FFA were not significantly different among the studied fish groups (Figure 4). The amount of Chol increased in group 2 and group 3 (experimental regimes) in comparison to group 1. The amount of sterol esters significantly decreased in groups 3 in comparison to group 1 and group 2. In October, TL was 32.7% in group 3 of underyearlings, 32.8% in group 1, and 34.2% dry weight in group 2 while CF was constant at 1.1%; no significant differences were found among the groups. A seasonal increase of TL was detected in underyearlings exposed to certain (same) photoperiod regimes (Table 2). It was found that the content of sterol esters decreased in group 3 in comparison to groups 1 and 2; along with this, the amount of total PL decreased in group 3 in comparison to group 2. The content of FFA increased in the experimental groups in comparision to control (Figure 4). Interestingly, the content of TL increased mainly due to TAG, major lipid class, from September to October in all studied groups.

**Figure 5:**
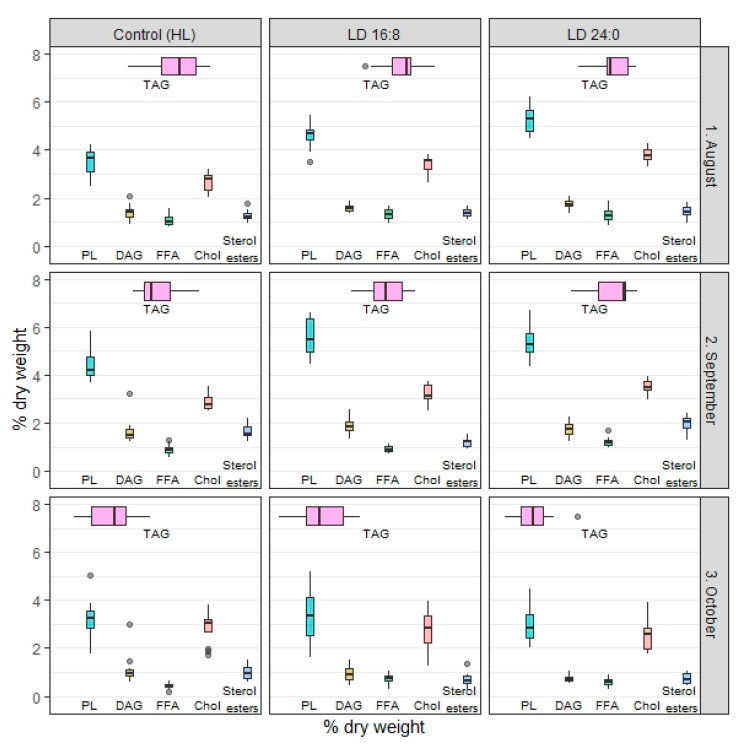
The changes in the lipid classes (% dry weight) in yearlings (at 0+ age) of Atlantic salmon (*Salmo salar* L.) under different photoperiod regimes: HL (hatchery lighting), the experimental regimes (LD 16:8, LD 24:0 from July 9 to October 5). The fish were exposed to different light regimes from July (fish was loaded into the tanks and the experiment was started); thus, the effect of photoperiod for yealings is discussed from August.

For yearlings, a significant seasonal decrease in TL content and CF for fish in group 1 and experimental groups 2 and 3 was found (Table 3). The effect of photoperiod on TL content of yearlings (control vs. experimental groups) was found in August and prolonged during the entire duration of the experiment. Recently [26], we published a detailed study on dynamic of lipid classes (including individual phospholipid fractions) in yearlings exposed to different photoperiods, including those discussed in the present study. Briefly, in August, the total content of PL, DAG, Chol and FFA increased in group 2 in comparison to group 1, and the total content of PL, DAG and Chol increased in group 3 in comparison to group 1 and group 2 (Figure 5). In September, as in August, the contents of total PL, DAG, Chol, FFA and sterol esters increased in experimental group 2 and group 3 in comparison to group 1 (control). In October, the content of TL decreased in all studied groups and the decline was the most significant in experimental group 3. It was found the decrease in the content of total PL, Chol and TAG in all studied groups. In the experimental groups, the amount of sterol esters and DAG was detected, while the content of FFA increased. It can be noticed that the content of TL due to TAG was depleted significantly in October.

### 3.3. Fatty Acids Profile

The changes in FAs profile in underyearlings under different photoperiod regimes are displayed in Table 4. The FAs profile in underyearlings (at 0+ age) was dominated by MUFAs (40.9–43.2% of total FA in the control, group 1 (hatchery light, HL) and the experimental group 2 (LD 16:8) and group 3 (LD 24:0)). The oleic FA (18:1n-9) was abundant (18.5–20.3% of total FAs); other MUFAs, such as 16:1(n-7), 18:1(n-7), 20:1(n-9), and 22:1(n-11), ranged from 3.0% to 6.3% of total FAs (Table 4). In September, total MUFA content in underyearlings showed no significant differences between group 1 and the experimental groups 2 and 3 (42.5–43.2% of total FAs), but individual FA varied. In group 2, 22:1(n-11) declined in September, while 18:1(n-7) and 22:1(n-11) increased in group 3. The above changes were minor, but differed from group 1. In October (vs. September), changes in the FA profile in underyearlings were significant in group 3. A significant decline in comparison to groups 1 and 3 were found in the content of dominating 18:1(n-9) and minor 22:1(n-11) vs. both LD 16:8 and LD 24:0 regimes (Table 4).

The total PUFAs accounted for 29.4–33.1% of the total FAs in underyearlings from September to October. The dominant family was (n-3) PUFA (21.0–24.8%), DHA accounted for the majority of the content: 13.1–15.2% of the total FAs. In September, we found no significant differences in the content of individual PUFAs in underyearlings subjected to the experimental light regime vs. the control. In October, in group 2 and especially in group 3, we observed an increase in total PUFA, mainly due to (n-3) PUFA, owing to physiologically valuable EPA and DHA being detected. The metabolic index 16:0/18:1(n-9) also increased.

The content of SFAs in underyearlings during the experiment (September–October) was within 26.0–27.4% of total FAs, and palmitic FA (16:0), prevailed among SFAs (16.0–17.1% of total FAs). Other SFAs did not exceed 4.5% of the total FA content. FA variations were observed in both total SFAs and their individual components in group 3; in September, SFAs content increased due to 14:0 (vs. groups 1 and 2). In October, the content declined at the expense of 18:0 (vs. group 3 in September).

The FA profile of total lipids in salmon underyearlings (at age 0+) and yearlings (at age 1+) reared under different light regimes in August, September, and October was dominated by MUFAs (within 44.2–48.4% of total FAs), where the highest proportion was 18:1(n-9) (26.4–30.34% of total FAs), while the other FAs did not exceed 6.0%. In August and September, group 3 exposure to LD 24:0 resulted in a significant increase in 18:1(n-7) in both age groups, and in October, group 3 experienced a decrease in total MUFAs at the expense of 18:1(n-9) (group 3 vs. group 1). The changes in FAs profile in yearlings (at 1+ age) under different photoperiod regimes are performed in Table 5.

In hatchery-reared yearlings, PUFAs contributed the second-largest percentage in total FA after MUFAs. Total PUFAs in yearlings in the August–October period constituted 27.9–34.0% of total FAs, with a 15.6–20.9% prevalence of (n-3) PUFA, and an elevated proportion of DHA (7.6–12.1% of total FAs). In August, group 3 yearlings exposed to LD 24:0 experienced a reduction (vs. control and group 2, LD 16:8) in total n-3 PUFAs (including DHA, EPA, and the minor 18:4(n-3)). In September, group 2, exposed to LD 16:8, demonstrated a reduction in 18:4(n-3), EPA, and DHA; in group 3 (LD 24:0), essential 18:2(n-6) and 18:3(n-3) FAs increased (all the changes compared with the matching light regimes in August). In October, yearlings, especially those in group 3 (LD 24:0), demonstrated a rise (vs. the control in October and vs. LD 24:0 in September) in the content of the physiologically valuable DHA, EPA, and ARA. An increase was observed in the (n-3)/(n-6) PUFA ratio, which portrays the ratio of the two PUFA families, and in the metabolic rate index 16:0/18:1(n-9).

During the August–October period, total SFAs content in yearlings was within 20.8–24.2% of total FA, which was lower than in underyearlings in September and October (26.0–27.4% of total FAs).

## 4. Discussion

### 4.1. Weight Gain in the Hatchery-Reared Underyearlings and Yearlings under Different Photoperiods

Among morphological indices, size indices (weight, length, condition factor) are highly important to smoltification, and at the hatchery the parameter is monitored and controlled for released hatchery-reared smolts of salmon, particularly with the aim of increasing adult returns. In this study, we mainly focus on weight gain, because mass is directly related to the volume of fish and correlates with lipid deposition and thus indicates the status of fish. Furthermore, it is known [27,28] that the size alone is a less valuable parameter for saltwater transfer of the Atlantic salmon and the stage of smoltification has to be obligatorily considered [29].

It was determined that the photoperiod (the studied prolonged and continuous light regimes) have different effects on the growth of underyearlings and yearlings. The highest weight gain was found for underyearlings reared under constant light. Moreover, the mortality rate was lower in experimental groups with additional lighting. It was shown [30] that constant light positively affects the growth rate and the aerobic and anaerobic capacities of the muscles of salmon underyearlings reared in a fish hatchery. The obtained results are in good accordance with other results obtained in previous studies [31,32]. The underyearlings exposed to LD 16:8 had lower mass at the end of the experiment. These results differed on other studies on Atlantic salmon [33,34], which demonstrated the best weight gain under this regime. It might be suggested that prolonged light (LD 16:8) is effective for growth in summer (summer season temperatures in the North freshwater bodies) and depends on the life stage of the fish [35].

In contrast, yearlings exposed to the experimental regimes LD 16:8 and LD 24:0 exhibited weight loss during the first two months. These results can be explained by the reaction to the new conditions and the necessity of an adaptation period. In September and October, the growth rate of fish from the HL group decreased, and yearlings from the experimental groups continued to grow. The artificial experimental light regimes made it possible to extend the period of fish active feeding, and consequently the yearlings in the experimental groups reached the weight of HL group. It is noteworthy that the parrs needed an adaptation period to new light conditions. It is possible that the period of additional light exposure should be longer.

### 4.2. The Total Lipid Content and Change of Lipid Classes and Fatty Acids in the Hatchery-Reared Underyearlings and Yearlings under Different Photoperiods

Previously, we accumulated baseline data on the lipid status of parr and smolts of Atlantic salmon from wild populations inhabiting freshwater in the Kola Peninsula [36,37,38,39]. Fatty acids are involved in an organism’s adaptive reactions in response to changes in the environment, participate in energy and physiological processes in the cell, and help regulate biochemical reactions. The contents and compositions of PUFAs and MUFAs in fish largely depend on the temperature and dietary regime, as well as on the organism’s ability to adjust the contents to the growth and development settings, as well as to the effects of external factors. In the majority of fish species, like in other living organisms, foraging and physiological activity rhythms are primarily governed by light cycles [1]. In the present research, we have shown the effects associated with changes in the TL content in the studied fish to be age-specific (physiology associated), as well as associated with different light regimes (external factors). The change of TL was maintained by the rearrangement of certain lipid classes. In this way, the increased content of Chol and total PL, in which we found that the content of phosphatidylcholine increased [26] in yearlings from the experimental groups, can be explained by the compensatory reaction to decrease the free radical oxidation (regulated by temperature and light) due to increase in the content of hard oxidized lipids. No doubt, the revealed changes in DAG, FFA, Chol and sterol esters in underyearlings and yearlings from the experimental groups indicate the involvement of these lipid classes in cell signaling and hormonal status modifications. We suggest that future research should be focused on the study of the effect of different photoperiod on the hormonal status of hatchery-reared young of Atlantic salmon.

The decline in total MUFAs at the expense of 18:1(n-9), observed in October in Atlantic salmon underyearlings and yearlings exposed to LD 24:0 (vs. the control), was positively correlated with a reduction in sterol esters (mainly Chol esters) storage in underyearlings, and triacylglycerols (TAG) and Chol esters in yearlings [26]. Storage lipids (TAG and Chol esters), which respond to changes in the external environment (temperature, photoperiod, etc.), provide an influx of energy for the activated physiological processes in fish as the rapidly mobilized (especially from TAG) FAs are oxidized. It is known that 18:1(n-9) accumulates in tissues rich in storage lipids, as the principal substrate for β-oxidation in salmonids [37,40,41]. MUFAs within storage lipids (TAG and Chol esters) are essential, and are the most readily available source of energy, promptly responding to changes in habitat. It is known that during the middle and late stages of smoltification of salmon, lipolysis and glycogenolysis activate to maintain energy-dependent parr–smolt transformation [42]. Ruchin [43] demonstrated that Siberian sturgeon *Acipenser baerii* exposed to 12, 16, and 24 h of light per day experienced an increase in respiration intensity, food consumption, and conversion, and their energy costs also increased. The October reductions in MUFAs, including 18:1(n-9), as well as minor 18:1(n-7), 20:1(n-9), and 22:1(n-11) in the juveniles, especially yearlings, were associated with exposure to continuous light (LD 24:0), and indeed with seasonal decrease in water temperature. However, studies on Baltic salmon [43] and Siberian sturgeon [43] showed that seasonal variations in their growth rate were mainly due to variations in daylight duration rather than water temperature.

In salmon underyearlings and yearlings in the hatchery-based experiments, the second largest share in total FAs after MUFAs belonged to the PUFAs, in contrast to the young growing and developing in natural fluvial habitats. Juvenile salmon from rivers of the Kola Peninsula contained more PUFAs [8,38,44,45].

The minor, yet visible increase in the physiologically valuable EPA and DHA, typical in marine fish, detected in October in underyearlings and yearlings exposed to LD 24:0, the rise in the 22:6(n-3)/18:3(n-3) and 20:4(n-6)/18:2(n-6) ratios (indicators of the conversion of essential 18:3(n-3) and 18:2(n-6) FA in longer-chain PUFA) in yearlings, and the rise in the FA metabolic rate indicator 16:0/18:1n-9 were positively correlated with a decrease in 18:1(n-9). Our own and other authors’ previous studies have demonstrated that oleic acid is actively metabolized in salmon parr as a source of energy, especially during smoltification [39,46,47]. The above-mentioned changes in the contents of EPA and DHA (which were the most pronounced in yearlings under the LD 24:0 treatment in October) are vitally important for juvenile fish and, as reported in the literature, are associated with hormonal transformations and regulation of nerve cells and the vision system [48,49]. Notably, the modifications of PUFA and their ratios in hatchery-reared salmon parr (yearlings at 1+ age) are comparable to those previously detected in wild parr (fish at 3+ age) salmon during their transformation into smolts [8], and can serve as markers of the organism’s physiological status. These modifications appear at an earlier age in juveniles reared under artificial 24 h light. The increase in marine-type specific EPA and DHA observed in October in underyearlings and yearlings salmon (especially under LD 24:0) suggests they were physiologically preparing for overwintering (both ages) and migration into the marine environment (mainly for yearlings, some of them showed silvering cover even late autumn), while wild young of salmon smoltify at different ages, but often later than 1+ [50,51,52]. The behavioral program aimed at smoltification, and downstream migration would be launched in young fish only after they had attained a certain weight and lipid status [15,50]. We have to point out here that in our recent study [38], the comparison of weight and length characteristics, as well as the content of DHA and EPA, and their metabolic precursors as 18:3(n-3) and 18:2(n-6) for hatchery-reared and wild underyearlings, showed that weight characteristics were higher for the former ones, while the ratio of essential 18:3(n-3)/18:2(n-6) was higher in wild fish due to differences in diets. Due to differences in diets, the technology of the hatchery aims to grow fish ready to smoltify in a shorter time, and the differences in DHA for underyearlings and yearlings between hatchery-reared and wild revealed that DHA was higher in hatchery-reared underyearlings and the content of FA was equal for both. Thus, the preparedness for smoltification is occurs in a shorter period of time for hatchery-reared underyearlings and yearlings.

## 5. Conclusions

The influence of two light regimes, 16:8 h light/dark (LD 16:8) and 24:0 h light/dark (LD 24:0), on the lipids and fatty acids content and weight gain in hatchery-reared underyearlings (at 0+ age) and yearlings (at 1+ age) of Atlantic salmon in the summer–autumn period was studied. It was found that the photoperiod (the studied prolonged and continuous light regimes) has different effects on the growth of underyearlings and yearlings. The highest weight gain was found for underyearlings reared under constant light. Both regimes of artificial experimental lighting prolonged the growth period of yearlings (1+) in comparison to fish reared under the control light regime (usual photoperiod at the hatchery). Along with the findings presented above, a seasonal increase of total lipids was found in underyearlings exposed to certain (same) photoperiod regimes. A seasonal decrease in total lipid content mainly due to nonpolar lipids (in October) was observed in yearlings under control and both experimental photoperiod regimes. The results indicate differences in structure- and energy-metabolism, with structure-metabolism (growth of tissues and organs) over fat accumulation (energy-metabolism) in yearlings exposed to experimental photoperiod. Moreover, the effect of photoperiod on total lipid content of yearlings (control vs. experimental groups) was found from the beginning of the experiment, and continued throughout the entire duration of the experiment. We have to point out that the mortality rate was lower in the experimental groups of underyearlings with additional light. Exposure to prolonged and continuous light did not affect yearlings’ mortality rate.

The increase in marine-type specific EPA and DHA observed in October in salmon underyearlings and yearlings (especially under LD 24:0) suggests they were physiologically preparing for overwintering. This also suggests that the observed elevated level of DHA in underyearlings might show the stimuli of the studied lighting on lipid metabolic processes of preparedness for smoltification even at such young age. The changes in fatty acids and their ratios in juvenile Atlantic salmon can be used as biochemical indicators of the degree to which hatchery-reared fish are ready to smoltify in comparison to fish at the same age from wild populations. Certainly, these include, first of all, an increase in marine-type specific DHA and EPA, an increase in the 16:0/18:1(n-9) ratio (an indicator of metabolic rate), in correlation with a reduction in MUFAs (mainly 18:1(n-9)), which are known to be actively metabolized by juveniles during smoltification. These biochemical modifications, accompanied by fish weight gain, were more pronounced in October in yearlings exposed to continuous light (LD 24:0). Thus, by experimentally selecting and manipulating light regimes, it is possible to control the vector of lipid metabolic processes in artificially reared salmon parr (age-specifically) toward marine-type transformation and coordinate the timing of smoltification and parr preparedness for release into the wild.

Moreover, the obtained results, in combination with other biochemical parameters (hormonal status, energy and carbohydrate metabolism, myogenesis, etc.), could improve the rearing process so as to significantly promote growth rate, natural resistance, adaptive capacity, and, eventually, the viability of the young and their readiness for release into the wild in the North. These findings are of practical value for correction juvenile salmon rearing techniques so they are ready to migrate to the sea, which is necessary for restoring natural Atlantic salmon populations.

## Figures and Tables

**Table 1:** Usual light regimes of the hatchery during the experimental period.

Time Period	July–10th of August	10th of August–10th of September	10th of September–the Entire October
Light regime	24 h Natural light regime Sunrise and sunset at the location of the Vygsky fish hatchery and certain latitude can be found here: https://sunsetsunrisetime.com/sun/belomorsk	from 8 a.m. till 5 p.m. Natural light regime from 5 a.m. till 8 p.m. The hatchery’s light was turned (artificial light)	24 h The hatchery’s light was turned (artificial light)

**Table 2:** Length-weight characteristics, condition factor (CF, %) and total lipids (% dry weight) in underyearlings (at 0+ age) of Atlantic salmon (Salmo salar L.) under different photoperiod regimes: HL (hatchery lighting), the experimental regimes—LD 16:8, LD 24:0 from August 5 to October 5. The effect of photoperiod on underyearlings was evaluated in September and in October (in August, the light regimes were installed and switched on).

Month	September (September 5)			October (October 5)		
Light regime	Control (HL)	LD 16:8	LD 24:0	Control (HL)	LD 16:8	LD 24:0
Group	1	2	3	1	2	3
n	20	20	20	20	20	20
Weight, g (average ----------- min-max)	2.5 ± 0.1 ------------ 1.9–3.1	2.5 ± 0.1 ----------- 1.9–3.0	2.6 ± 0.2 ----------- 1.6–4.3	3.7 ± 0.0 ^A^ ------------ 3.0–4.7	3.5 ± 0.1 ^A^ ------------ 2.6–4.9	3.7 ± 0.03 ^A^ ------------ 2.5–5.5
Length, cm (average ----------- min-max)	6.1 ± 0.1 ----------- 5.8–6.5	6.1 ± 0.1 ----------- 5.5–6.4	6.2 ± 0.1 ----------- 5.4–7.3	7.2 ± 0.1 ^A^ ------------ 6.7–7.7	7.0 ± 0.1 ^A^ ------------ 6.4–7.9	7.1 ± 0.1 ^A^ ------------ 6.2–8.2
Condition factor (CF, %)	1.1 ± 0.0	1.1 ± 0.0	1.1 ± 0.0	1.0 ± 0.0 ^A^	1.0 ± 0.0 ^A^	1.0 ± 0.0 ^A^
TL, % dry weight	27.1 ± 0.3	26.8 ± 0.9	26.6 ± 0.4	32.8 ± 0.9 ^A^	34.2 ± 0.8 ^A^	32.7 ± 0.9 ^A^

Note: ^A^—the difference is significant (*p* ≤ 0.05) compared to fish exposed same light regime in September; ^1^—the difference is significant (*p* ≤ 0.05) compared to fish from the control, group 1; ^2^—the difference is significant (*p* ≤ 0.05) compared to fish exposed to LD 16:8, group 2.

**Table 3:** Length-weight characteristics, condition factor (CF, %) and total lipids (% dry weight) in yearlings (at 1+ age) of Atlantic salmon (*Salmo salar* L.) under different photoperiod regimes: HL (hatchery light regime), the experimental regimes (LD 16:8, LD 24:0 from July 9 to October 5). The fish were exposed to different light regimes from July (fish were loaded into the tanks and the experiment was started); thus, the effect of photoperiod for yealings is discussed from August.

Month	August (August 8)			September (September 5)			October (October 5)		
Light regime	Control (HL)	LD 16:8	LD 24:0	Control (HL)	LD 16:8	LD 24:0	Control (HL)	LD 16:8	LD 24:0
Group	1	2	3	1	2	3	1	2	3
Weight, g (average ----------- min-max)	21.6 ± 0.8 ----------- 16.4–25.8	16.6 ± 0.8 ^1^ ------------ 10.8–20.5	19.1 ± 0.5 ^1,2^ ------------- 15.1–22.2	26.7 ± 0.4 ^A^ ------------ 15.5–39.7	26.7 ± 0.4 ^A^ -------------- 17.6–37.7	25.3 ± 0.3 ^A^ ------------- 18.2–31.3	28.1 ± 0.3 ^A^ -------------- 16.9–53.0	27.5 ± 0.6 ^A^ ------------- 19.5–44.1	29.6 ± 0.3 ^A^ ------------- 22.0–41.8
Length, cm (average ----------- min-max)	11.9 ± 0.1 ----------- 11.0–12.6	11.0 ± 0.2 ^1^ ------------- 9.5–12.0	11.1 ± 0.1 ^1^ ------------- 10.5–11.8	13.0 ± 0.3 ^A^ ------------ 10.7–15.1	13.0 ± 0.2 ^A^ ------------ 11.3–14.4	12.8 ± 0.2 ^A^ ------------ 11.3–13.9	13.5 ± 0.1 ^A^ ------------- 11.4–16.2	13.4 ± 0.2 ^A^ ------------ 11.9–15.7	13.8 ± 0.1 ^AB^ --------------- 12.6–15.4
Condition factor (CF)	1.3 ± 0.0	1.3 ± 0.0	1.4 ± 0.2 ^1,2^	1.2 ± 0.0 ^A^	1.2 ± 0.0 ^A^	1.2 ± 0.0 ^A^	1.1 ± 0.0 ^AB^	1.1 ± 0.0 ^AB^	1.1 ± 0.0 ^AB^
TL, % dry weight	26.7 ± 1.0	29.5 ± 0.8 ^1^	30.0 ± 0.7 ^1^	26.2 ± 0.8	28.6 ± 0.9 ^1^	30.2 ± 0.8 ^1^	18.6 ± 1.1 ^AB^	17.9 ± 1.3 ^AB^	15.9 ± 1.0 ^AB1^

Note: ^A^—the difference is significant (*p* ≤ 0.05) compared to fish exposed same light regime in August; ^B^—the difference is significant (*p* ≤ 0.05) compared to fish exposed same light regime in September; ^1^—the difference is significant (*p* ≤ 0.05) compared to fish from the control, group 1; ^2^—the difference is significant (*p* ≤ 0.05) compared to fish exposed to LD 16:8, group 2.

**Table 4:** Fatty acid composition (% of total FA) in underyearlings (at 0+ age) of Atlantic salmon (*Salmo salar* L.) under different photoperiod regimes: HL (hatchery lighting), the experimental regimes (LD 16:8, LD 24:0 from August 5 to October 5). The effect of photoperiod on underyearlings was evaluated in September and in October (in August, the light regimes were installed and switched on).

Month	September (September 5)			October (October 5)		
Light regime	Control (HL)	LD 16:8	LD 24:0	Control (HL)	LD 16:8	LD 24:0
Group	1	2	3	1	2	3
Fatty acids	% of total FA					
14:0	4.2 ± 0.0	4.1 ± 0.0	4.4 ± 0.1 ^1,2^	4.3 ± 0.2	4.1 ± 0.0	4.2 ± 0.1
16:0	16.4 ± 0.1	16.4 ± 0.1	17.1 ± 0.3	16.8 ± 0.6	16.0 ± 0.1 ^A^	16.1 ± 0.1
18:0	3.9 ± 0.1	4.0 ± 0.0	4.1 ± 0.1	3.9 ± 0.1	3.8 ± 0.1	3.7 ± 0.0 ^A^
∑ SFA	26.5 ± 0.1	26.4 ± 0.1	27.4 ± 0.5	27.0 ± 0.9	26.1 ± 0.2	26.0 ± 0.2 ^A^
16:1(n-7)	5.3 ± 0.1	5.2 ± 0.0	5.1 ± 0.1	5.3 ± 0.2	5.1 ± 0.0	5.1 ± 0.0
18:1(n-9)	19.7 ± 0.5	20.3 ± 0.4	19.7 ± 0.4	19.9 ± 0.6	19.6 ± 0.2	18.5 ± 0.3 ^1,2^
18:1(n-7)	3.0 ± 0.0	3.3 ± 0.2	3.2 ± 0.0 ^1^	3.4 ± 0.1 ^A^	3.1 ± 0.0 ^1^	3.4 ± 0.2
20:1(n-9)	5.3 ± 0.1	5.2 ± 0.1	5.5 ± 0.1	5.5 ± 0.2	5.2 ± 0.1	5.3 ± 0.1
22:1(n-11)	6.1 ± 0.1	6.0 ± 0.0 ^1^	6.3 ± 0.1^2^	5.7 ± 0.2	5.6 ± 0.1 ^A^	5.7 ± 0.1 ^A^
∑ MUFA	42.5 ± 0.4	43.2 ± 0.3	43.1 ± 0.7	43.0 ± 1.4	41.6 ± 0.1 ^A^	40.9 ± 0.3 ^A1^
18:2(n-6)	5.5 ± 0.1	5.5 ± 0.1	5.3 ± 0.2	5.3 ± 0.1	5.4 ± 0.1	5.5 ± 0.0 ^2^
20:4(n-6)	0.5 ± 0.0	0.5 ± 0.0	0.5 ± 0.0	0.5 ± 0.0	0.5 ± 0.0 ^A^	0.6 ± 0.0 ^A^
∑ (n-6) PUFA	7.8 ± 0.2	7.5 ± 0.0	7.3 ± 0.2	7.5 ± 0.1	7.5 ± 0.1	7.7 ± 0.0 ^2^
18:3(n-3)	1.1 ± 0.0	1.1 ± 0.0	1.1 ± 0.0	1.1 ± 0.1	1.2 ± 0.0	1.2 ± 0.0 ^A^
18:4(n-3)	1.5 ± 0.1	1.4 ± 0.0	1.4 ± 0.1	1.4 ± 0.1	1.4 ± 0.0	1.5 ± 0.0 ^A2^
20:5(n-3)	3.4 ± 0.2	3.0 ± 0.1	2.9 ± 0.2	3.1 ± 0.3	3.4 ± 0.1 ^A^	3.5 ± 0.1 ^A2^
22:5(n-3)	1.3 ± 0.0	1.3 ± 0.0	1.2 ± 0.1	1.3 ± 0.1	1.4 ± 0.0 ^A^	1.4 ± 0.0
22:6(n-3)	13.1 ± 0.2	13.6 ± 0.2	13.1 ± 0.5	13.2 ± 1.4	14.9 ± 0.2 ^A^	15.2 ± 0.3 ^A^
∑ (n-3) PUFA	21.8 ± 0.4	21.8 ± 0.2	21.0 ± 0.9	21.4 ± 2.2	23.7 ± 0.2 ^A^	24.4 ± 0.4 ^A^
∑ PUFA	31.0 ± 0.5	30.4 ± 0.2	29.4 ± 1.2	30.0 ± 2.3	32.3 ± 0.3 ^A^	33.1 ± 0.4 ^A^
Others *	8.3	8.0	7.9	8.2	8.2	8.1
∑ (n-3)/∑ (n-6)	2.8 ± 0.1	2.9 ± 0.02	2.9 ± 0.1	2.8 ± 0.3	3.2 ± 0.0 ^A^	3.2 ± 0.1 ^A^
16:0/18:1(n-9)	0.8 ± 0.0	0.8 ± 0.02	0.9 ± 0.0 ^2^	0.8 ± 0.0	0.8 ± 0.0	0.9 ± 0.0 ^2^

Note: * The samples also contained 36 fatty acids each of which contributed not more than 1% to total FA, these FAs are not included in the table, but the summed amount of these FAs is presented. ^A^—the difference is significant (*p* ≤ 0.05) compared to fish exposed same light regime in September; ^1^—the difference is significant (*p* ≤ 0.05) compared to fish from the control, group 1; ^2^—the difference is significant (*p* ≤ 0.05) compared to fish exposed to LD 16:8, group 2.

**Table 5:** Fatty acid composition (% of total FA) in yearlings (at 1+ age) of Atlantic salmon (*Salmo salar* L.) under different photoperiod regimes: HL (hatchery lighting), the experimental regimes (LD 16:8, LD 24:0 from July 9 to October 5). The fish were exposed to different light regimes from July (fish were loaded into the tanks and the experiment was started); thus, the effect of photoperiod for yealings is discussed from August.

Month	August (August 8)			September (September 5)			October (October 5)		
Light regime	Control (HL)	LD 16:8	LD 24:0	Control (HL)	LD 16:8	LD 24:0	Control (HL)	LD 16:8	LD 24:0
Group	1	2	3	1	2	3	1	2	3
Fatty acids	% of total FA								
14:0	3.4 ± 0.1	3.4 ± 0.1	3.4 ± 0.1	3.1 ± 0.1 ^A^	3.0 ± 0.1 ^A^	3.0 ± 0.0 ^A^	2.8 ± 0.1 ^A^	2.7 ± 0.1 ^A^	2.7 ± 0.1 ^AB^
16:0	15.0 ± 0.4	14.7 ± 0.3	15.2 ± 0.3	13.8 ± 0.3	13.4 ± 0.4	13.2 ± 0.2 ^A^	13.6 ± 0.4 ^A^	13.0 ± 0.3 ^A^	13.5 ± 0.2 ^A^
18:0	3.6 ± 0.1	3.5 ± 0.1	3.8 ± 0.0 ^2^	3.4 ± 0.1	3.5 ± 0.1	3.3 ± 0.0 ^A^	3.3 ± 0.1	3.3 ± 0.1	3.6 ± 0.1 ^B^
∑ SFA	23.8 ± 0.6	23.5 ± 0.4	24.2 ± 0.4	21.8 ± 0.5 ^A^	21.4 ± 0.6	21.0 ± 0.0 ^A^	21.5 ± 0.5 ^A^	20.8 ± 0.6 ^A^	21.7 ± 0.3 ^A^
16:1(n-7)	4.2 ± 0.2	4.3 ± 0.1	4.5 ± 0.2	4.0 ± 0.1	3.8 ± 0.1 ^A^	3.8 ± 0.1 ^A^	3.6 ± 0.1 ^A^	3.5 ± 0.1 ^A^	3.4 ± 0.1 ^AB^
18:1(n-9)	26.7 ± 0.9	26.4 ± 0.6	28.3 ± 1.1	29.6 ± 0.8	30.0 ± 0.8 ^A^	30.3 ± 0.4	29.6 ± 0.2	30.0 ± 0.3^A^	28.2 ± 0.6 ^B2^
18:1(n-7)	3.4 ± 0.1	3.3 ± 0.0	3.6 ± 0.1 ^2^	3.3 ± 0.0	3.4 ± 0.0	3.4 ± 0.0 ^1^	3.3 ± 0.1	3.2 ± 0.0 ^B^	3.1 ± 0.1 ^A^
20:1(n-9)	4.5 ± 0.1	4.6 ± 0.1	4.7 ± 0.1	4.8 ± 0.1	4.7 ± 0.1	4.8 ± 0.0	4.5 ± 0.2	4.4 ± 0.1	4.2 ± 0.1 ^AB^
22:1(n-11)	3.9 ± 0.1	4.0 ± 0.1	4.1 ± 0.1	3.9 ± 0.1	3.8 ± 0.2	3.7 ± 0.1 ^A^	3.5 ± 0.2	3.5 ± 0.1 ^A^	3.2 ± 0.1 ^AB^
∑ MUFA	45.3 ± 0.5	45.2 ± 0.3	47.9 ± 0.7 ^1,2^	48.0 ± 0.5 ^A^	48.0 ± 0.5 ^A^	48.4 ± 0.3	46.9 ± 0.4	46.8 ± 0.2 ^A^	44.2 ± 0.5 ^AB1,2^
18:2(n-6)	9.5 ± 0.3	9.5 ± 0.3	9.3 ± 0.3	10.1 ± 0.3	10.4 ± 0.2	10.5 ± 0.1 ^A^	10.0 ± 0.2	10.5 ± 0.3	10.0 ± 0.2 ^B^
20:4(n-6)	0.4 ± 0.0	0.4 ± 0.0	0.4 ± 0.0 ^2^	0.4 ± 0.0	0.4 ± 0.0	0.4 ± 0.0	0.4 ± 0.0	0.5 ± 0.0 ^AB^	0.6 ± 0.0 ^B1^
∑ (n-6) PUFA	11.8 ± 0.4	11.8 ± 0.3	11.5 ± 0.3	12.4 ± 0.3	12.7 ± 0.3	12.9 ± 0.1 ^A^	12.4 ± 0.3	12.8 ± 0.3	12.4 ± 0.2 ^A^
18:3(n-3)	2.3 ± 0.1	2.3 ± 0.1	2.2 ± 0.1	2.6 ± 0.2	2.6 ± 0.1	2.8 ± 0.0 ^A^	2.6 ± 0.1	2.8 ± 0.2	2.6 ± 0.1
18:4(n-3)	1.3 ± 0.0	1.3 ± 0.0	1.1 ± 0.0 ^1,2^	1.2 ± 0.0 ^A^	1.2 ± 0.0	1.2 ± 0.0	1.0 ± 0.1 ^A^	1.0 ± 0.1	1.0 ± 0.0 ^B^
20:5(n-3)	2.8 ± 0.1	2.8 ± 0.1	2.3 ± 0.2 ^2^	2.4 ± 0.1	2.3 ± 0.1 ^A^	2.3 ± 0.1	2.4 ± 0.1	2.6 ± 0.0 ^B^	2.8 ± 0.2 ^B1^
22:5(n-3)	1.2 ± 0.1	1.2 ± 0.0	1.1 ± 0.1	1.1 ± 0.0	1.1 ± 0.0 ^A^	1.1 ± 0.1	1.0 ± 0.1	1.0 ± 0.0	1.1 ± 0.1
22:6(n-3)	9.3 ± 0.3	9.6 ± 0.3	7.6 ± 0.5 ^1,2^	8.5 ± 0.3	8.7 ± 0.3	8.4 ± 0.2	10.2 ± 0.2	10.1 ± 0.2 ^B^	12.1 ± 0.3 ^AB2^
∑ (n-3) PUFA	18.2 ± 0.4	18.6 ± 0.4	15.6 ± 0.7 ^1.2^	17.0 ± 0.3 ^A^	17.1 ± 0.4 ^A^	17.0 ± 0.3	18.5 ± 1.6	18.8 ± 0.4 ^B^	20.9 ± 0.5 ^AB1,2^
∑ PUFA	30.8 ± 0.4	31.3 ± 0.3	27.9 ± 0.5 ^1,2^	30.1 ± 0.2	30.5 ± 0.5	30.6 ± 0.3 ^A^	31.6 ± 1.8	32.4 ± 0.7	34.0 ± 0.3 ^AB^
Others *	7.6	7.8	6.9	7.0	6.9	7.1	7.5	7.1	7.1
∑ (n-3)/∑ (n-6)	1.6 ± 0.1	1.6 ± 0.1	1.4 ± 0.1	1.4 ± 0.1	1.4 ± 0.1	1.3 ± 0.0	1.5 ± 0.1	1.5 ± 0.0 ^B^	1.7 ± 0.1 ^AB2^
16:0/18:1(n-9)	0.6 ± 0.0	0.6 ± 0.0	0.5 ± 0.0	0.5 ± 0.0	0.5 ± 0.0 ^A^	0.4 ± 0.0 ^A^	0.5 ± 0.0 ^A^	0.4 ± 0.0 ^A^	0.5 ± 0.0 ^B1^

Note: * The samples also contained 36 fatty acids, each of which contributed no more than 1% to total FA, these FAs are not included in the table, but the summed amount of these FAs is presented. ^A^—the difference is significant (*p* ≤ 0.05) compared to fish exposed same light regime in August; ^B^—the difference is significant (*p* ≤ 0.05) compared to fish exposed same light regime in September; ^1^—the difference is significant (*p* ≤ 0.05) compared to fish from the control, group 1; ^2^—the difference is significant (*p* ≤ 0.05) compared to fish exposed to LD 16:8, group 2.
